# Giant basal cell carcinoma of the back in an elderly lady—excision and OZ plasty

**DOI:** 10.1093/jscr/rjaf589

**Published:** 2025-08-20

**Authors:** Ananth S Mathad, Rajiv Sindhu Shivmani, Akhil Palod, Naveena A N Kumar

**Affiliations:** Department of Surgical Oncology, Manipal Comprehensive Cancer Care Center, Kasturba Medical College, Manipal, Karnataka 576104, India; Manipal Academy of Higher Education, Manipal, Madhava Nagar, Karnataka 576104, India; Department of Surgical Oncology, Manipal Comprehensive Cancer Care Center, Kasturba Medical College, Manipal, Karnataka 576104, India; Manipal Academy of Higher Education, Manipal, Madhava Nagar, Karnataka 576104, India; Department of Surgical Oncology, Manipal Comprehensive Cancer Care Center, Kasturba Medical College, Manipal, Karnataka 576104, India; Manipal Academy of Higher Education, Manipal, Madhava Nagar, Karnataka 576104, India; Department of Surgical Oncology, Manipal Comprehensive Cancer Care Center, Kasturba Medical College, Manipal, Karnataka 576104, India; Manipal Academy of Higher Education, Manipal, Madhava Nagar, Karnataka 576104, India

**Keywords:** giant basal cell carcinoma, OZ plasty, wide local excision

## Abstract

Giant basal cell carcinoma is a rare type of basal cell carcinoma, which is distinguished by its propensity for early metastases and deep tissue penetration. Here, we describe a case of 76-year-old woman who had a large ulceroinfiltrative lesion on her lower back that was diagnosed as basal cell carcinoma pre-operatively. Following a wide local excision with a negative margin, an OZ plasty flap was used for immediate reconstruction. Postoperative recovery was uneventful, and the 1-year follow-up revealed no recurrence and excellent functional and cosmetic outcomes. In older patients with few reconstructive choices, this case study demonstrates that OZ plasty is a viable, and cosmetically good reconstructive technique.

## Introduction

Giant basal cell carcinoma (GBCC) is an uncommon variant of basal cell carcinoma (BCC) with an aggressive nature and propensity for extra-dermal t invasion to underlying bone and muscle. The biological behaviour of GBCC is pugnacious and shows a high metastasis and recurrence rate [1]. According to the American Joint Committee on Cancer (AJCC), GBCC is a dermatological malignancy with clinical-histopathological features of BCC with a diameter larger than 5 cm. GBCC with a size of more than 10 cm has a 45% chance of fatal outcome, and these tumours are infrequently reported in the literature with 0.5%–1% incidence [1, 2].

GBCC is frequently encountered on the back, followed by the face and upper limbs. Patients usually notice the pathology late due to its location. It shows a high incidence in elderly male patients, usually in seventh decade of life. The natural history is of a long-standing cutaneous tumour with a typical duration of 14.5 years [2, 3]. There are no established treatment guidelines for GBCC, but the mainstay for its treatment remains aggressive surgical excision with a negative margin followed by local flap cover or split skin grafting.

We report a case of GBCC of the back. The patient underwent wide local excision and immediate OZ plasty for defect reconstruction. On follow-up, the flap had a satisfactory cosmetic outcome, and in our opinion, it can be used as a technically feasible and cosmetically sound reconstructive option in such cases.

## Case report

**Figure 1:**
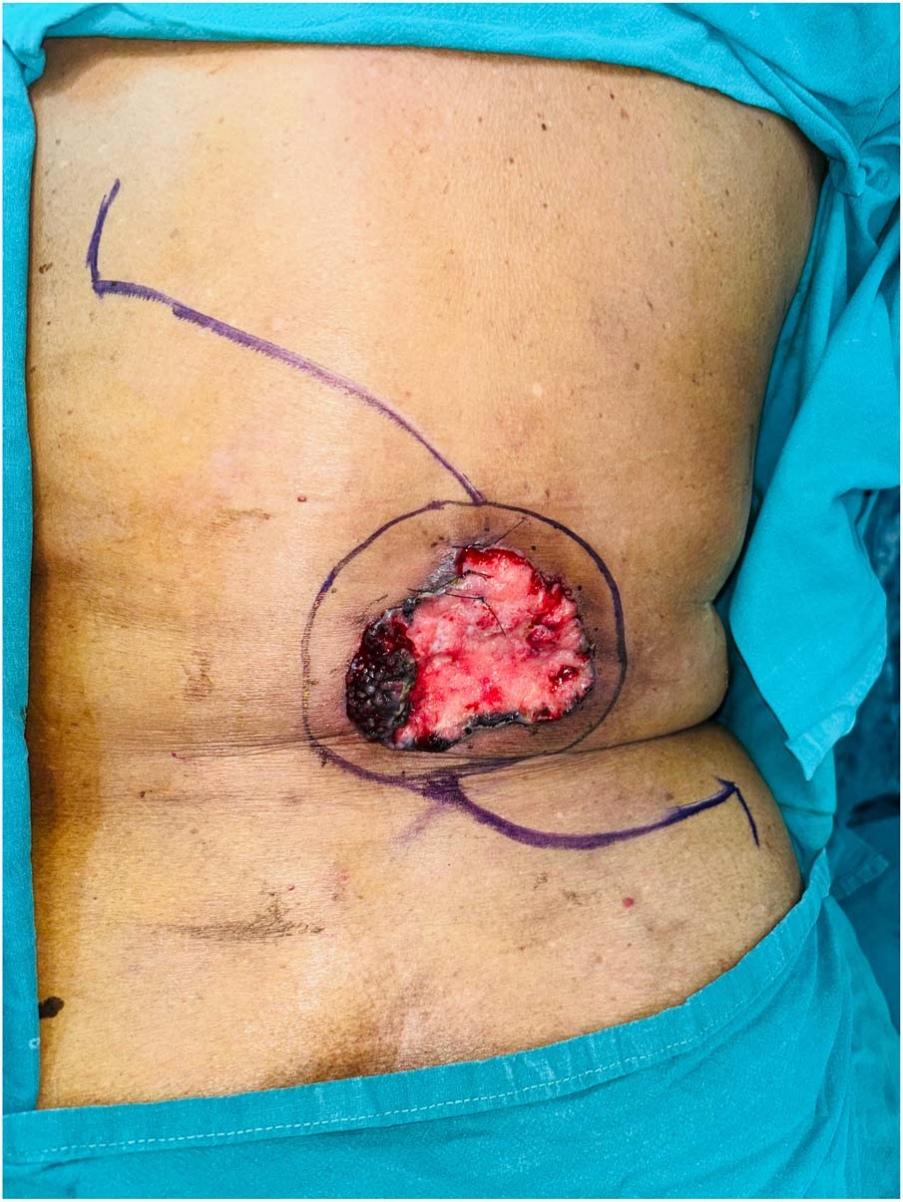
Preoperative GBCC of the back measuring 10 × 10 cm lesion with flap marking.

We present the case of a 76-year-old elderly woman who presented with a pigmented lesion on her lower back for 2 years. She had multiple comorbidities (type 2 diabetes, hypertension, ischemic heart disease). On examination, a 10 × 10 cm irregular, pigmented, ulceroinfiltrative lesion was noted on the lower back (Fig. 1). A biopsy performed at a local hospital suggested BCC.

**Figure 2:**
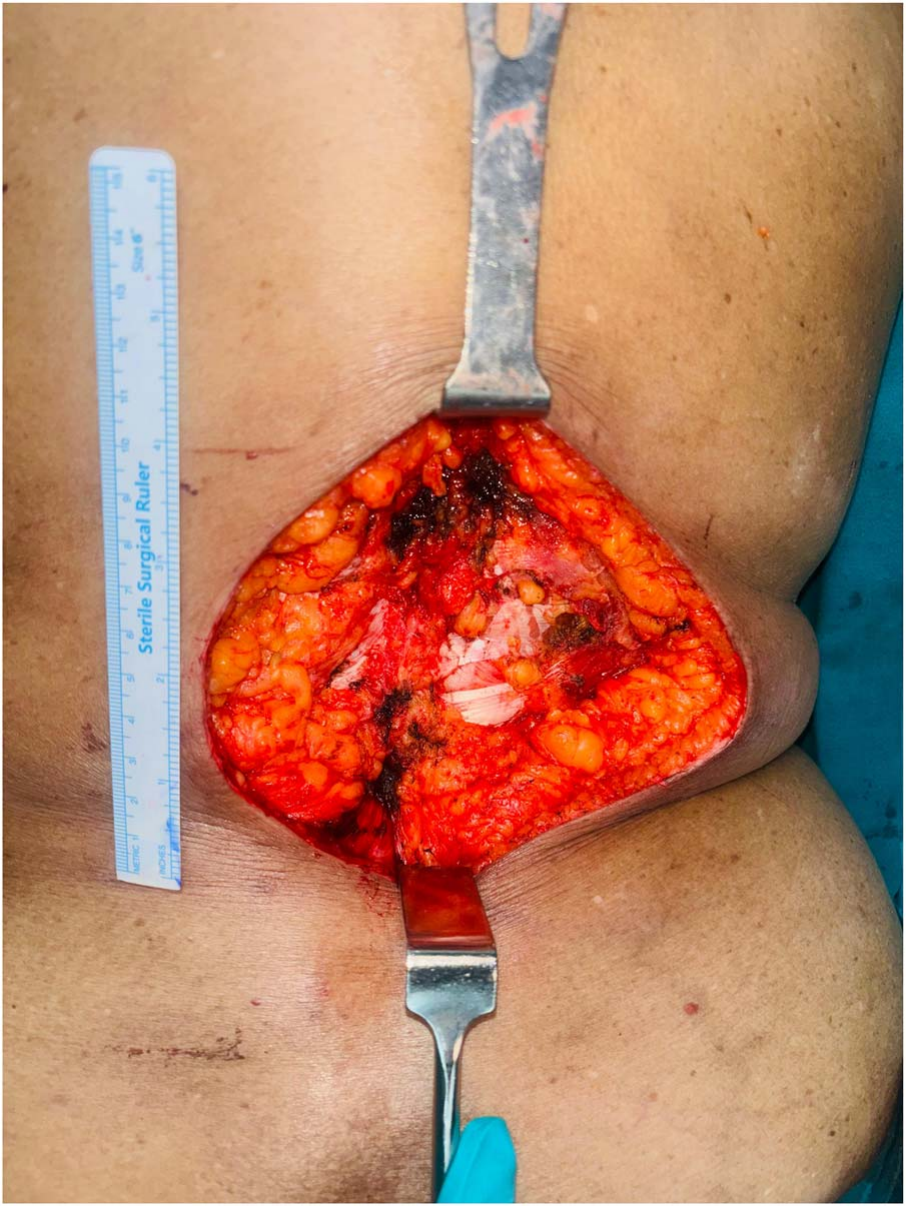
Intraoperative figure showing the defect after tumour excision—defect size 13 × 12 cm.

**Figure 3:**
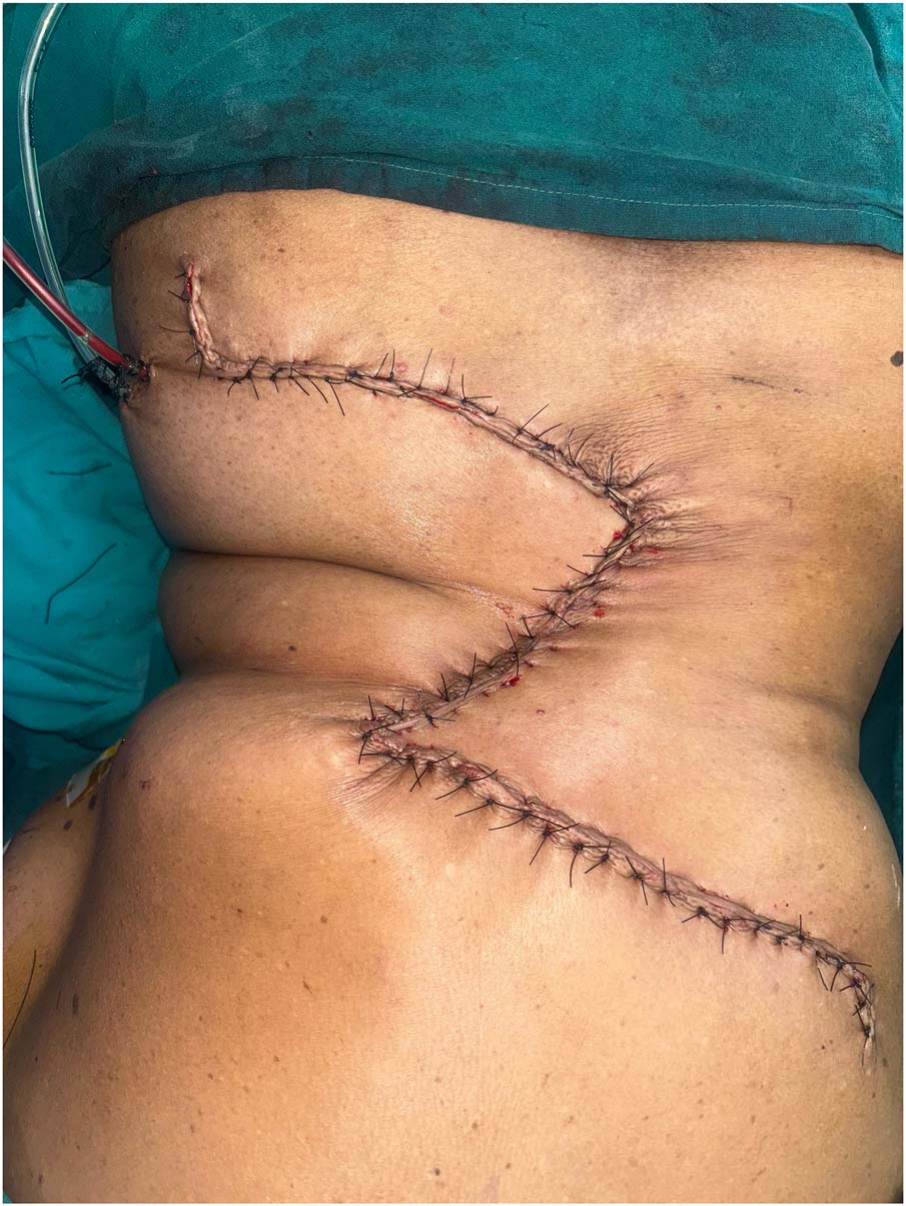
Immediate postoperative result after O-Z flap reconstruction.

Preoperative punch biopsy confirmed BCC and imaging with computed tomography (CT) scan demonstrated lesion confined to skin and subcutaneous fat with no evidence of disease elsewhere including locoregional lymph nodes. Following a multidisciplinary tumour board discussion, she underwent wide local excision with 1 cm margin (Fig. 2) up to the skeletal muscle and immediate reconstruction by OZ plasty for defect coverage (Fig. 3), as other flap reconstruction was not feasible in view of comorbidities and generalized atherosclerosis noticed on imaging. Postoperative period was uneventful. Histopathology confirmed BCC, pTNM-pT3 with high-risk features, including a tumour size of 8 cm, a depth of invasion of 5 mm, Clark’s Level IV, and surface ulceration. No lymphovascular or perineural invasion was observed, and microscopic margins were free. She was planned for close observation after discussing in tumour board.

**Figure 4:**
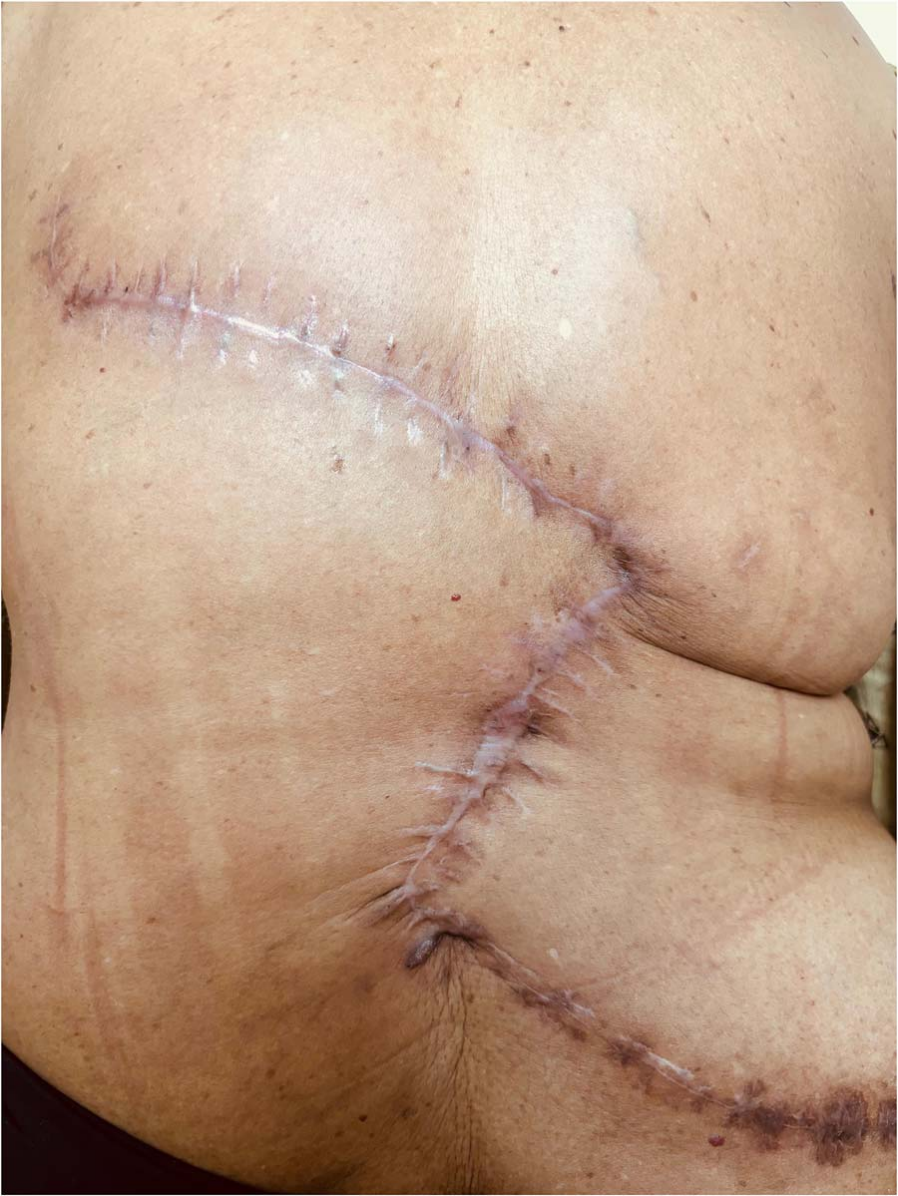
Follow-up picture showing a healthy scar.

On regular follow-up and at the end of 1 year, the surgical site was healthy with no functional or cosmetic issues (Fig. 4).

## Discussion

GBCCs are nefarious skin tumours with a proclivity for early and more profound extradermal tissue invasion and metastasis. A GBCC, according to AJCC, is deficient as BCC with a diameter of more than 5 cm and becomes stage T3 in TNM staging for cutaneous malignancy [2]. In the clinicopathological case review by Archontaki *et al*., the most common GBCC variant was the nodular type, followed by the infiltrating and superficial types, respectively [1].

Hypoalbuminemia, chronic anaemia, history of burns, alcohol abuse, and radiation exposure are common risk factors for the development of GBCC. In the study by Archontaki *et al*., these tumours were found to have a 9.8% recurrence rate [1]. Due to the large diameter of these tumours, their increased predisposition for distant metastasis, deeper tissue infiltration, wide excision and extensive bony, and soft tissue reconstruction are challenging [1, 4, 5].

The surgical aim in GBCC is to perform wide local excision with a microscopic negative margin, single-stage reconstruction. The site, defect size, and the underlying structures exposed after the wide local excision determine the reconstruction technique used. Depending on the abovementioned factors, the reconstruction can involve free flaps, local transpositional, rotational local flaps, or grafting.

Lackey *et al*. reported a series of eight cases, one of which involved a patient undergoing a chest wall reconstruction using a transverse rectus abdominis muscle (TRAM) flap [6]. In a case series of 48 cases of GBCC, Archontaki *et al*. found 13 cases of back GBCCs with a single case describing a myocutaneous flap for reconstruction. They also described one back reconstruction with a bilateral myocutaneous latissimus dorsi flap and a Fascio cutaneous gluteal flap in their personal series of three cases [1].

Bogdanić *et al*. reported a case report of GBCC of the back treated by wide local excision and skin grafts [7]. A case report of GBCC of the back was reported by Sinha *et al*., where the patient underwent an excision followed by reconstruction with a double perforator flap [4].

Complex reconstructive involving pedicled free flap and microvascular anastomosis may be required in cases of GBCC of the back due to a dearth of local tissue for coverage. Due to advanced age and underlying comorbidities, these patients are not suitable for complex reconstructive techniques. Despite its excellent extensive defect coverage ability, split skin grafting may not be ideal over deep wounds with exposed bone. In such patients, local flap using OZ plasty is an outstanding choice. OZ plasty is a simple reconstruction technique with no need for microsurgical equipments and techniques. Care must be taken during preoperative planning of reconstruction to prevent dog-ear deformity, flap necrosis, tension-free tissue mobilization, and local vascularity. It has a short learning curve and provides a well-vascularized flap over the defect with good cosmetic and functional outcomes.

## Conclusion

GBCC of the back is an aggressive dermal malignancy that demands an aggressive surgical treatment in the form of margin-negative wide local excision and simultaneous reconstruction. Defect coverage may be challenging due to the lack of local soft tissue and the inability to perform complex reconstruction because of surgical and anaesthetic fitness, especially in elderly. OZ plasty is a simple, effective, and cosmetically sound choice of reconstruction in such cases where other reconstructive techniques may not be feasible.
